# SR Protein Kinase 1 Inhibition by TAF15

**DOI:** 10.3390/cells12010126

**Published:** 2022-12-28

**Authors:** Anastasia Koukiali, Makrina Daniilidou, Ilias Mylonis, Thomas Giannakouros, Eleni Nikolakaki

**Affiliations:** 1 Laboratory of Biochemistry, Department of Chemistry, Aristotle University, 54124 Thessaloniki, Greece; 2 Department of Neurobiology, Care Sciences and Society, Division of Neurogeriatrics, Center for Alzheimer Research, Karolinska Institutet, 10435 Stockholm, Sweden; 3 Laboratory of Biochemistry, Faculty of Medicine, University of Thessaly, Biopolis, 41500 Larissa, Greece

**Keywords:** SRPK1, TAF15, RGG motif, SR proteins, LBR, nucleolin, HNRNPU, HNRNPA2B1

## Abstract

Although SRPKs were discovered nearly 30 years ago, our understanding of their mode of regulation is still limited. Regarded as constitutively active enzymes known to participate in diverse biological processes, their prominent mode of regulation mainly depends on their intracellular localization. Molecular chaperones associate with a large internal spacer sequence that separates the bipartite kinase catalytic core and modulates the kinases’ partitioning between the cytoplasm and nucleus. Besides molecular chaperones that function as anchoring proteins, a few other proteins were shown to interact directly with SRPK1, the most-studied member of SRPKs, and alter its activity. In this study, we identified TAF15, which has been involved in transcription initiation, splicing, DNA repair, and RNA maturation, as a novel SRPK1-interacting protein. The C-terminal RGG domain of TAF15 was able to associate with SRPK1 and downregulate its activity. Furthermore, overexpression of this domain partially relocalized SRPK1 to the nucleus and resulted in hypophosphorylation of SR proteins, inhibition of splicing of a reporter minigene, and inhibition of Lamin B receptor phosphorylation. We further demonstrated that peptides comprising the RGG repeats of nucleolin, HNRPU, and HNRNPA2B1, were also able to inhibit SRPK1 activity, suggesting that negative regulation of SRPK1 activity might be a key biochemical property of RGG motif-containing proteins.

## 1. Introduction

The SRPK subfamily of protein kinases is highly conserved throughout eukaryotes, from Caenorhabditis elegans to humans, thus rendering these molecules’ central importance in cellular function apparent [1]. Even though SRPKs are widely considered splice factor kinases due to their decisive involvement in regulating various steps of mRNA splicing, they were also shown to affect diverse cellular activities by phosphorylating multiple serine residues residing in consecutive arginine-serine dipeptide repeats, known as RS domains [1,2,3,4]. Given that the mammalian genome contains more than a hundred RS domain-containing proteins [4,5], their pleiotropic mechanism of action might be attributed not only to the phosphorylation of diverse substrates but also to the different extents of phosphorylation of their substrates. In this way, their mode of action is more akin to a rheostat than a binary switch, thus transducing changes in the phosphorylation state into changes in protein function.

Most of our knowledge regarding SRPKs comes from studies on SRPK1. *SRPK1* is an essential gene for embryonic mouse development, since knocking it out results in a lethal phenotype [6]. SRPK1′s function is mainly controlled by its intracellular localization. In interphase cells, SRPK1 localizes predominantly in the cytoplasm, with more or less faint staining in the nucleus [7,8]. A large non-conserved spacer region that separates the two catalytic kinase domains was shown to associate with the Hsp70/90 chaperone complexes, thus anchoring SRPK1 to the cytoplasm [8]. Cytoplasmic SRPK1 phosphorylates newly synthesized SR proteins and facilitates their nuclear import via a specific member of the importin-beta family, transportin-SR2 [9,10]. Besides SR proteins, SRPK1 phosphorylates other proteins in the cytoplasm, like protamine 1 [11], ZO-2 [12] and RNF12 [4], and also promotes their transport to the nucleus. The “steady-state” cytoplasmic localization of SRPK1 is altered by external signals, such as growth factors [13], genotoxic agents [14], osmotic stress [8] and cell cycle signals [7,15], that dissociate the kinase from the chaperone complexes, resulting in its translocation to the nucleus. In general, it is believed that a growth factor/hormone-mediated increase in the nuclear concentration of SRPK1 may alter the phosphorylation level of SR proteins and, consequently, their activity on their primary transcript targets, favoring the expression of splicing isoforms that contribute to cell growth. On the contrary, nuclear accumulation of the kinase after deletion of the spacer region or after treatment of cells with genotoxic agents is closely related to inhibition of growth (see ref. [16] for a review).

Even though SRPK1 has been considered constitutively active [17], two post-translational modifications have been reported to modulate its activity. First, the disulfide bonding of cysteine residues stabilizes a loop-like structure of the spacer region, thus bringing the two catalytic domains into proximity, which is a prerequisite for kinase activity [18,19]. Second, phosphorylation of SRPK1 by CK2 at Ser51 results in an approximately 6-fold activation of the kinase in vitro [20]. In addition, several proteins were shown to modulate SRPK1-mediated phosphorylation either by interacting with the kinase and blocking its activity or by associating with the substrates’ RS domains, thus masking the phosphorylation sites. Specifically, binding of the nuclear scaffold proteins SAFB1 and SAFB2 to SRPK1 was shown to repress its activity [21], while during herpes simplex virus infection, SRPK1 is inactivated by interaction with the viral protein ICP27 [22,23]. Furthermore, the multi-functional cellular protein p32 has been reported to bind to the unmodified RS domains of ASF/SF2 and Lamin B Receptor (LBR) and prevent their phosphorylation [24,25].

**Figure 1:**
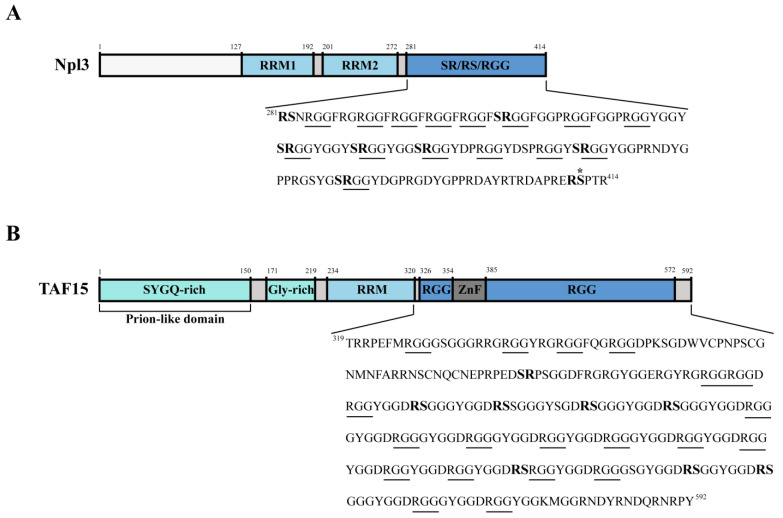
Domain structure and sequence of the RS/RGG domain of *S. cerevisiae* Npl3 (**A**) and human TAF15 (**B**). RGG repeats and SR/RS dipeptides are underlined and in bold, respectively.

TAF15 belongs to a family of proteins known as FET, including FUS and EWSR1. These proteins bind to RNA and are implicated in central cellular processes such as transcription, splicing, and RNA transport [26]. TAF15 is closely related to the yeast protein Npl3 (NCBI, Ensembl, Protein Atlas), which is also an RNA-binding protein involved in mRNA splicing, mRNA export, and translation. Sky1, the yeast orthologue of SRPK1, was reported to phosphorylate Npl3p at its C-terminus [27]. Npl3′s C-terminal RGG domain contains eight dispersed SR/RS dipeptides, only the final of which (marked with an asterisk in Figure 1) was found to be phosphorylated by Sky1 [27]. TAF15′s C-terminal RGG domain also contains eight dispersed SR/RS dipeptides (Figure 1).

In an attempt to identify novel functions of SRPK1, we initially sought to test whether TAF15, similarly to Npl3, could act as a substrate of the kinase. We present evidence that while the C-terminal RGG domain of TAF15 was able to interact with SRPK1, it was not phosphorylated by the kinase; on the contrary, this interaction resulted in SRPK1 inhibition. Significantly, overexpression of this domain resulted in hypophosphorylation of SR proteins, inhibition of splicing of a reporter minigene, and inhibition of LBR phosphorylation. These data support a new role for TAF15 as an inhibitor of SRPK1 function. Moreover, peptides comprising the RGG repeats of three proteins—nucleolin, HNRPU, and HNRNPA2B1—previously found to co-immunoprecipitate with SRPK1a, an alternatively spliced isoform of SRPK1 [28], were also able to inhibit SRPK1 activity. Hence, RGG domain-mediated negative regulation of SRPK1 might be an important novel mechanism for modulating SRPK1 function.

## 2. Materials and Methods

### 2.1. Plasmids and Expression of Proteins 

pGEX-2T-SRPK1, pFLAG-CMV-2-SRPK1, pGEX-2T-LBRNt(62–92) (expressing a 31-amino-acid fragment of the N-terminal domain of LBR that contains the RS dipeptides), and pEGFP-C1-TAF15(319–589) have been previously described [29,30,31]. To subclone TAF15(319–589), hereby referred to as TAF15-RGG, into pGEX-2T, the respective cDNA was amplified with PCR from pEGFP-C1-TAF15-RGG with the upstream primer 5′-TGGATCCACTAGAAGACCTGAATTCATGAGAGGAGGT-3′ and downstream primer 5′-TCCCGGGGTATGGTCGGTTGCGCTGATCAT-3′ and ligated into the BamHI/SmaI site of pGEX-2T. pVP16-TAF15 expressing full-length TAF15 with an 8xHis tag fused to its N-terminus, was purchased from Arizona State University, USA. TAF15 was amplified with PCR from pVP16-TAF15 with the upstream primer 5′- CGGATCCATGTCGGATTCTGGAAGTTACGGTCAG-3′ and downstream primer 5′-TCCCGGGGTATGGTCGGTTGCGCTGATCAT-3′ and ligated into the BamHI/SmaI site of pGEX-2T. To subclone TAF15(1–320) into pGEX-2T, the respective cDNA was amplified with PCR from pVP16-TAF15 with the upstream primer 5′-CGGATCCATGTCGGATTCTGGAAGTTACGGTCAG-3′ and the downstream primer 5′-TCCCGGGTCTAGTGGCAAAGGACACTTTAATGATGTTG-3′ and ligated into the BamHI/SmaI site of pGEX-2T. The GST- and His- fusion proteins were produced in bacteria and purified using glutathione-Sepharose (Amersham Biosciences GmbH, Freiburg, Germany) and nickel chromatography (Qiagen GmbH, Hilden, Germany) respectively, according to the manufacturer’s instructions. 

### 2.2. Cell Culture and Transfection

HeLa cells were cultured in DMEM medium supplemented with 10% (*v/v*) fetal bovine serum (FBS) and antibiotics (cell culture products were purchased from Gibco-Invitrogen, Waltham, MA, USA). Cells were incubated at 37 °C with 5% CO_2_. Transfections of plasmids expressing GFP alone or GFP-TAF15-RGG were done with the Xfect™ transfection kit (Clontech-Takara Bio, Mountain View, CA, USA) according to the manufacturer’s instructions. Briefly, 4 × 10^4^ HeLa cells were plated in 24-well plates, and 1 μg of plasmid DNA was diluted with Xfect Reaction Buffer to a final volume of 50 μL and added to 0.25 mL DMEM (without FBS). Following 4 h of incubation, nanoparticle complexes were removed via aspiration, and 1 mL of fresh complete growth medium was added. Cells were collected after 48 h. For immunoprecipitation experiments, transfections with 10 μg of plasmids expressing GFP alone or GFP-TAF15-RGG were carried out with jetPRIME (PolyPlus, Illkirch, France) according to the manufacturer’s instructions.

### 2.3. Pull-Down Assays and Immunoprecipitation: Detection of Phosphorylated SR Proteins

Incubation of GST and GST-SRPK1 (2–3 μg each) immobilized on glutathione-Sepharose beads with His-TAF15 (2 μg) was performed in 20 mm Hepes, pH 7.5, 50 mM NaCl, and 1% Triton X-100 in a total volume of 0.5 mL. Similar incubations were also performed in the presence of 150 and 300 mM NaCl to test the stringency of the interaction. The incubations were carried out for 60 min at room temperature. The beads were harvested, washed three times with 500 μL of binding buffer, and resuspended in 25 μL of SDS sample buffer. Bound His-TAF15 was analyzed on 10% SDS-polyacrylamide gels and detected by Coomassie Blue staining. 

Incubation of GST, GST-TAF15, GST-TAF15(1–320), and GST-TAF15-RGG (2–3 μg each) immobilized on glutathione-Sepharose beads with HeLa cell extracts (∼200 μg of total protein) was performed in 50 mm Tris-HCl, pH 7.5, 150 mm NaCl, 1% Triton X-100, and 0.5 mm PMSF (cell extraction buffer) in a total volume of 0.5 mL for 60 min at room temperature. Bound SRPK1 was analyzed on 10% SDS-polyacrylamide gels and detected by Western blotting using the anti-SRPK1 monoclonal antibody (611072, BD Biosciences, San Jose, CA, USA), an alkaline phosphatase-coupled goat anti-mouse secondary antibody, and a 5-bromo-4-chloro-3-indolyl phosphate/nitro blue tetrazolium substrate.

For immunoprecipitation experiments, HeLa cells transfected with GFP or GFP-TAF15-RGG were lysed for 30 min on ice in 25 mM Hepes, pH 7.4, 300 mM NaCl, 2 mM MgCl_2_, 2% Triton X-100, and 0.5 mM PMSF to extract nuclear envelope proteins. Cell extracts were clarified by centrifugation at 13,000 rpm for 15 min in a microcentrifuge, and the supernatants were diluted with 25 mM Hepes pH 7.4, 2 mM MgCl_2_, and 0.5 mM PMSF to a final concentration of 25 mM Hepes pH 7.4, 150 mM NaCl, 2 mM MgCl_2_, 1% Triton X-100, and 0.5 mM PMSF. LBR was immunoprecipitated from diluted extracts using an anti-LBR monoclonal antibody (GTX61107, GeneTex, Irvine, CA, USA). Immunoprecipitates were washed three times with the diluted buffer, resuspended in 25 μL of SDS sample buffer, and analyzed on 10% SDS-polyacrylamide gels. Phosphorylated LBR was detected by Western blotting using an anti-P-Ser polyclonal antibody (AB-1603, Merck Millipore, Billerica, MA, USA).

For the detection of phosphorylated SR proteins, Western blot analysis of similarly diluted extracts (∼100 μg of total protein) was performed with the mAb104 monoclonal antibody (kindly provided by Prof. Jamal Tazi, IGMM, CNRS, Université de Montpellier, France). Western blotting images were taken by a Uvitec Cambridge Chemiluminescence Imaging System (Uvitec Cambridge, Cambridge, UK) using Alliance software, version 16.06, and quantified by Uviband Software. 

### 2.4. In Vitro Kinase Assays

Kinase assays were carried out at 30 °C in a total volume of 25 μL containing 12 mM Hepes pH 7.5, 10 mM MgCl_2_, 25 μM ATP, 1–3 μg of the appropriate substrate (GST-LBRNt(62–92) or GST-TAF15 or His-TAF15), and 1–2 μg GST-SRPK1. For the inhibition assays, GST-SRPK1 was incubated with GST-LBRNt(62–92) under the assay conditions in the presence of increasing concentrations of GST-TAF15, GST-TAF15-RGG, and GST-TAF15(1–320) or Nucl, SAFA, and A2B1 peptides, as indicated. Nucl (RGGGRGGFGGRGGGRGGRGGFGGRGRGGFGGRGGFRGGRGG), SAFA (RGGGHRGRGGFNMRGGNFRGGAPGNRGGY) and A2B1 (RGGNFGFGDSRGGGGNFGPGPGSNFRGG) peptides, comprising the RGG repeats of nucleolin, HNRPU (SAFA), and HNRNPA2B1, respectively, were provided by GeneCust, Boynes, France. The samples were incubated for 30 min, and the reaction was stopped by adding 6 μL of 5 × SDS sample buffer and heating at 95 °C for 3 min. Phosphoproteins were detected via autoradiography using Super RX (a Fuji medical X-ray film), and signals were quantified by excising the radioactive bands from the gel and scintillation counting. *p*-values were determined using a two-tailed, unpaired student’s t-test.

### 2.5. Immunofluorescence Microscopy

HeLa cells transfected with plasmids encoding GFP-TAF15-RGG were grown on glass coverslips for 48 h. After the incubation period, the cell coverslips were fixed with 4% paraformaldehyde in phosphate-buffered saline (PBS) for 20 min at room temperature, and excess aldehyde was quenched with 100 mM Tris-HCl pH 7.5. Cells were then permeabilized with 0.2% Triton X-100 in PBS for 10 min and blocked with 0.5% fish skin gelatin (FSG) in PBS for 30 min. Probing with the primary (goat polyclonal anti-GFP diluted 1:1000, SICGEN Antibodies; mouse monoclonal anti-SRPK1 diluted 1:150, BD Biosciences, San Jose, CA, USA) and secondary (Alexa 488 donkey anti-goat diluted 1:350, Abcam; Alexa 488 donkey anti-mouse diluted 1:400, Invitrogen; TRITC-conjugated goat anti-mouse, diluted 1:800, Molecular Probes) and DNA staining (propidium iodide) were performed as previously described [18]. After three washes, the coverslips were mounted with a mounting medium (0.01% p-phenylenediamine and 50% glycerol in PBS) and visualized with a Nikon confocal microscope using the EZ-C1 3.20 software.

### 2.6. Splicing Reporter Minigene Assay

Splicing assays were performed as previously described [18] using the pSVIRB vector, which contains rat insulin exons 1, 2, and 3 and the respective introns, flanked by SV40 promoter/enhancer regions and insulin transcription terminators [32]. Briefly, HeLa cells (3 × 10^4^) were co-transfected with 1.2 μg of the reporter gene and increasing amounts (0.4, 0.8, and 1.2 μg) of plasmid DNA encoding GFP-TAF15-RGG, without or with also co-transfecting 0.4 μg of plasmid encoding FLAG-SRPK1. RNA was isolated 24 h following transfection using the RNeasy mini kit (Qiagen), and 1μg of it was reverse transcribed using the M-MLV RT kit (Invitrogen), according to the manufacturer’s instructions. Part of this reaction mixture (1 μL) was diluted to a final volume of 50 μL, the concentrations of the buffer and dNTPs were adjusted for PCR, and the mixture was initially denaturated at 94 °C for 2 min and then amplified for 30 cycles using Taq DNA polymerase, recombinant (Invitrogen). PCR conditions were: denaturation at 94 °C for 30 s, annealing at 65 °C for 1 min, extension at 72 °C for 1 min and a final extension at 72 °C for 5 min. The primers used were INS1 (sense): 5′-CAGCTACAGTCGGAAACCATCAGCAAGCAG-3′ and INS3 (antisense): 5′-CACCTCCAGTGCCAAGGTCTGAAGGTCACC-3′. Amplified products were resolved by electrophoresis through 1% agarose gel and ethidium bromide staining. The sizes of the unspliced and spliced products were ~1 and ~0.3 kb, respectively.

## 3. Results

### 3.1. TAF15 Is Not a Substrate of SRPK1

**Figure 2:**
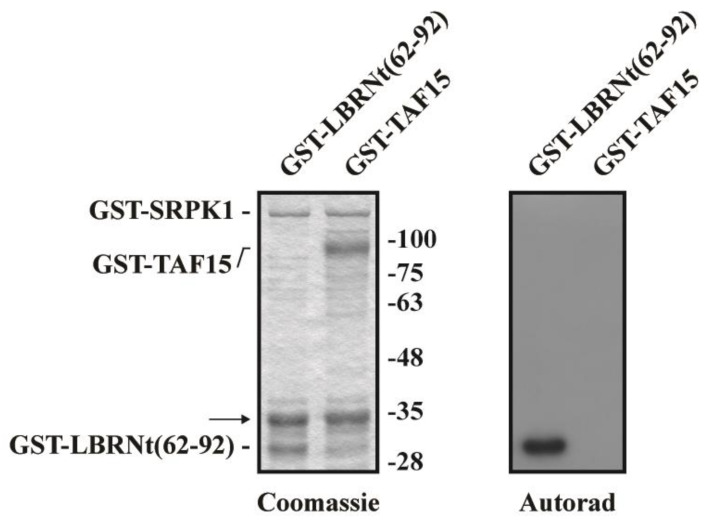
TAF15 is not a substrate of SRPK1. Phosphorylation of GST-LBRNt(62–92) and GST-TAF15 by GST-SRPK1. The band indicated by the arrow represents a GST-SRPK1 degradation product. Left panel, Coomassie blue staining; right panel, auto-radiography.

Based on the homology of the TAF15 C-terminal RGG domain with the respective domain of budding yeast Npl3 protein, we asked whether one or more of the dispersed SR/RS dipeptides present in this domain could be phosphorylated by SRPK1, similarly to Npl3p. To this purpose, we initially performed in vitro phosphorylation assays using bacterially produced GST-SRPK1 and GST-TAF15 as substrates. As shown in Figure 2, SRPK1 did not modify TAF15, whereas it efficiently phosphorylated GST-LBRNt(62–92), a well-characterized substrate of the kinase [31]. SRPK1 was also unable to phosphorylate full-length TAF15 with an 8xHis tag fused to its N-terminus (data not shown).

### 3.2. The C-terminal RGG Domain of TAF15 Binds to SRPK1 In Vitro

**Figure 3:**
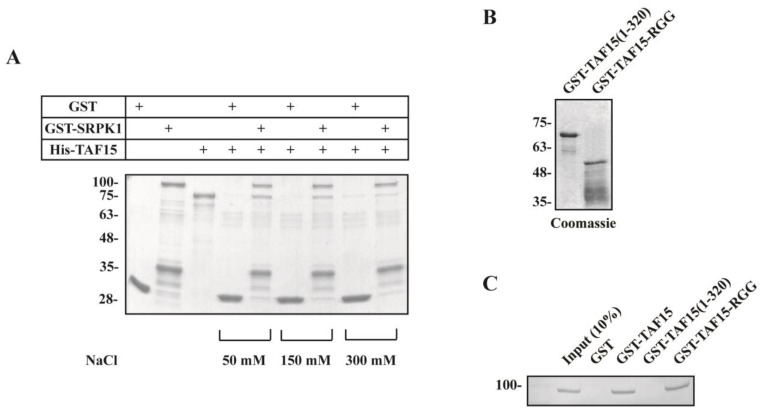
The C-terminal RGG domain of TAF15 interacts with SRPK1. (**A**) Pull-down assays using His-TAF15 and GST-SRPK1 in the presence of 50, 150, and 300 mM NaCl. GST was included as a control. Bound His-TAF15 was detected by Coomassie blue staining. (**B**) SDS-PAGE analysis and Coomassie blue staining of bacterially produced GST-TAF15(1–320) and GST-TAF15-RGG. The full-length GST-TAF15-RGG migrates with an apparent molecular mass of approximately 53 kDa. The lower bands represent degradation products. (**C**) GST, GST-TAF15, GST-TAF15(1–320), and GST-TAF15-RGG immobilized on glutathione-Sepharose beads were incubated with HeLa cell extracts. The sediments were harvested, washed three times with cell extraction buffer, and analyzed by SDS-PAGE. Bound SRPK1 was detected by Western blotting using the anti-SRPK1 monoclonal antibody. Numbers on the left indicate molecular masses (in kDa).

Even though TAF15 is not a substrate of SRPK1, we set out to investigate the potential interaction of these two proteins. To this end, we used in vitro pull-down assays to test the ability of full-length His-tagged TAF15 to bind GST-SRPK1 immobilized on glutathione-Sepharose beads. Bound His-TAF15 was analyzed by SDS-PAGE and Coomassie Blue staining. As shown in Figure 3A, TAF15 binds SRPK1 at physiological ionic strength (50–150 mM NaCl), while increased salt concentration (0.3 M NaCl) reduced binding. In addition, GST-TAF15 immobilized on glutathione-Sepharose beads, when incubated with HeLa cell extracts, was able to interact with SRPK1 (Figure 3C). To identify the region of TAF15 that was responsible for the observed interaction, we expressed the N-terminal part (aa 1–320) and the C-terminal RGG domain (aa 319–589) of TAF15 as GST-fusion proteins (Figure 3B). GST-TAF15(1–320) and GST-TAF15-RGG were then immobilized on glutathione-Sepharose beads and incubated with HeLa cell extracts. As shown in Figure 3C, GST-TAF15-RGG could bind to SRPK1, whereas no such interaction was obtained with GST-TAF15(1–320), suggesting that the binding was confined to the C-terminal RGG domain of TAF15.

### 3.3. The C-terminal RGG Domain of TAF15 Inhibits SRPK1 Activity In Vitro

**Figure 4:**
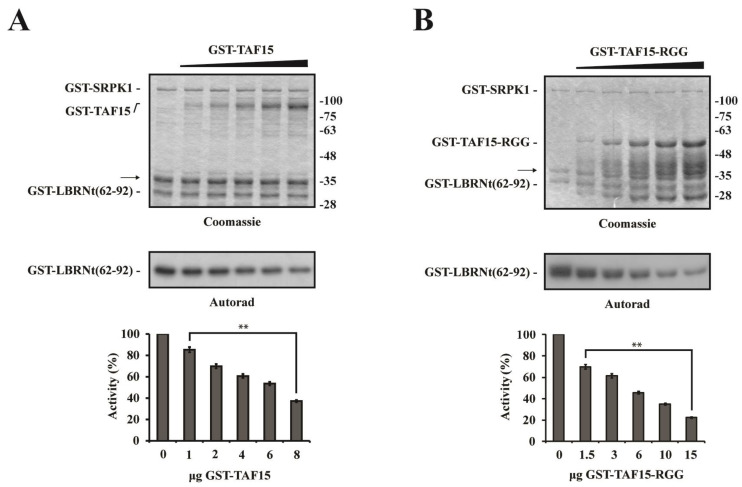
The C-terminal RGG domain of TAF15 inhibits SRPK1 activity. The GST-SRPK1 kinase was incubated with GST–LBRNt(62–92) and [γ-32P]ATP in the presence of 1, 2, 4, 6, and 8 μg GST-TAF15 (**A**) and 1.5, 3, 6, 10, and 15 μg GST-TAF15-RGG (**B**). The samples were analyzed by SDS-PAGE, Coomassie blue stained (upper panels), and autoradiographed (middle panels, only the respective part of the gel is shown). The band indicated by the arrow represents a GST-SRPK1 degradation product. The full-length GST-TAF15-RGG migrates with an apparent molecular mass of approximately 53 kDa. The lower bands represent degradation products. Phosphorylated bands were excised from the dry gel, and Cherenkof counted (lower panels). Data represent the means ± SE of two independent experiments. *p* values: ** *p* = 0.001063 (for GST-TAF15), ** *p* = 0.00812 (for GST-TAF15-RGG).

To determine whether the interaction of TAF15 with SRPK1 had any effect on the kinase activity, we performed in vitro phosphorylation assays using bacterially produced GST-LBRNt(62–92) as substrate in the presence of increasing amounts of GST-TAF15. As shown in Figure 4A, GST-TAF15 inhibited the phosphorylation of GST-LBRNt(62–92) in a dose-responsive manner. To define the region of TAF15 that was responsible for the observed inhibition, we repeated the phosphorylation assays in the presence of increasing concentrations of GST-TAF15-RGG and GST-TAF15(1–320). TAF15-RGG inhibited SRPK1 as potently as full-length TAF15 (Figure 4B), whereas TAF15(1–320) did not affect the kinase activity (Supplementary Figure S1), suggesting that the inhibitory activity resides within the C-terminal RGG domain of TAF15.

### 3.4. The C-terminal RGG Domain of TAF15 Alters SRPK1 Localization

**Figure 5:**
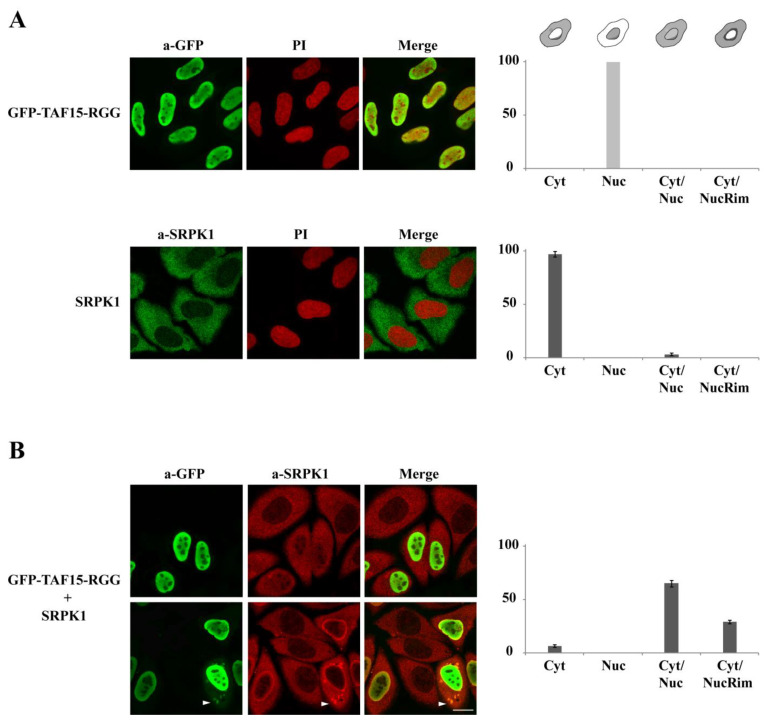
The C-terminal RGG domain of TAF15 relocalizes SRPK1 to the nucleus. (**A**) Fluorescent pattern of GFP-TAF15-RGG and endogenous SRPK1 in HeLa cells transfected with pEGFP-C1-TAF15-RGG or non-transfected cells, respectively. GFP-TAF15-RGG and SRPK1 were detected using the polyclonal anti-GFP and the anti-SRPK1 monoclonal antibodies, while nuclei were stained with PI. Scale bar: 10 µM. A diagrammatic representation of GFP and SRPK1 staining patterns is shown in the right panel. The percentage of GFP (light grey) and SRPK1 (dark grey) staining patterns relative to the indicated staining pattern was determined for 120 (GFP) and 200 (SRPK1) cells in two different experiments, where the means ± standard errors of the measurements are shown. (**B**) Fluorescent pattern of GFP-TAF15-RGG and endogenous SRPK1 in HeLa cells transfected with pEGFP-C1-TAF15-RGG. GFP-TAF15-RGG and SRPK1 were detected as before, using the polyclonal anti-GFP and the anti-SRPK1 monoclonal antibodies. Yet, in this case, the secondary Alexa 488 donkey anti-goat antibody was used to visualize GFP (green), and the secondary TRITC-conjugated goat anti-mouse antibody was used to visualize SRPK1 (red). Scale bar: 10 µM. The percentage of SRPK1 staining patterns (dark grey) relative to the indicated staining pattern was determined for 160 transfected cells in two replicates, where the means ± standard errors of the measurements are shown (**right panel**). Non-transfected cells that did not show any green fluorescence were not taken into consideration.

The association of TAF15 with SRPK1 and the subsequent decrease in kinase activity seems puzzling at first sight since TAF15 is a nuclear RNA-binding protein [30], while SRPK1 is predominantly cytoplasmic [7,8,14,18]. Since both the binding and inhibitory activity reside within the C-terminal RGG domain of TAF15 and the nuclear localization sequence (NLS) of TAF15 resides in its C-terminal 32 amino acid residues [33], we sought to test whether overexpression of TAF15-RGG would influence the localization of SRPK1. In this respect, immunofluorescent staining of GFP-TAF15-RGG in HeLa cells was exclusively nuclear, while SRPK1 was, as expected, mainly present in the cytoplasm of non-transfected cells (Figure 5A). However, when we examined SRPK1 localization in HeLa cells transfected with GFP-TAF15-RGG, we noticed that a fraction of SRPK1 relocalized to the nucleus (Figure 5B). Specifically, in only 6% of the transfected cells, the cytoplasmic localization of SRPK1 remained unaffected, while in 65% of the transfected cells, diffuse staining of SRPK1 throughout the nucleus was also observed, and in the remaining 29% of the cells, a fraction of SRPK1 showed clear nuclear rim fluorescence (Figure 5B, right panel). Careful examination of the staining suggested that a diffuse nuclear localization of GFP-TAF15-RGG mostly correlated with a similar diffuse nuclear staining of SRPK1. When the fluorescence of GFP-TAF15-RGG was more intense at the nuclear periphery, a significant localization of SRPK1 to the nuclear rim was also observed (Figure 5B, lower panel). In a few cells, some co-localization of GFP-TAF15-RGG with SRPK1 in cytoplasmic foci that could represent cytoplasmic stress granules was observed (Figure 5B, indicated with an arrow).

The observed relocalization of SRPK1 was highly reminiscent of that seen by ICP27 in HSV-1-infected cells [22]. In these cells, ICP27 showed either diffuse nuclear staining or strong nuclear rim fluorescence and caused analogous relocalization of SRPK1 [22].

### 3.5. Overexpression of the C-terminal RGG Domain of TAF15 Alters the Splicing of a Reporter Gene

**Figure 6:**
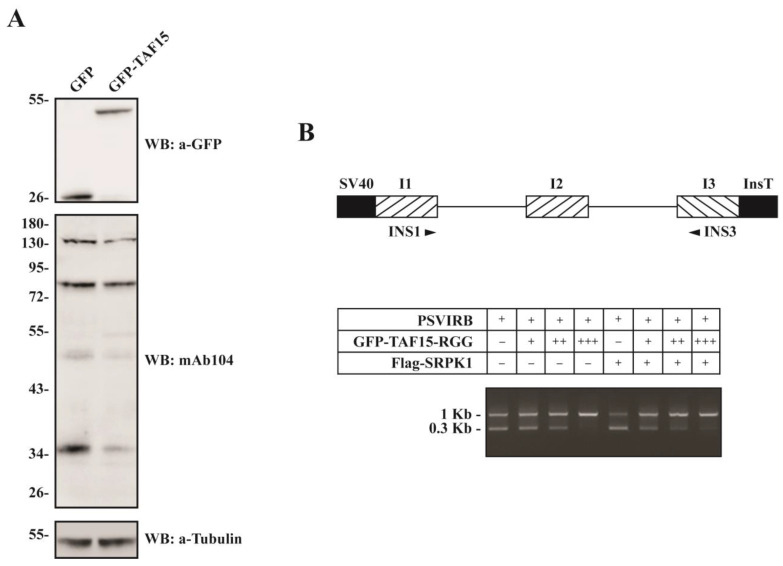
Overexpression of TAF15-RGG results in reduced SR protein phosphorylation and alters the splicing of a reporter minigene. (**A**) mAb104 analysis of the phosphorylation state of SR proteins from HeLa cells transfected with GFP or GFP-TAF15-RGG. The expression levels of GFP and GFP-TAF15-RGG are shown in the upper panel, while immunoblotting with a mouse monoclonal anti-tubulin antibody was used as a loading control (**lower panel**). Numbers on the left indicate molecular masses (in kDa). (**B**, **upper panel**) Schematic representation of the insulin expression construct. SV40 promoter/enhancer and transcription terminator regions are shown in black, exons (I1, I2, and I3) are diagonally striped, and introns are indicated as lines. The primers used for PCR are indicated by arrows. (**B**, **lower panel**) HeLa cells were transfected with 1.2 μg of the reporter gene along with increasing concentrations (0.4, 0.8, and 1.2 μg) of pEGFP-C1-TAF15-RGG (indicated as +, ++, and +++, respectively) without (first four lanes) or with (last four lanes) co-transfecting 0.4 μg of plasmid encoding FLAG-SRPK1. RNA was isolated 24 h later, and RT-PCR was carried out. The sizes of the spliced and unspliced products are 290 bp and ~1kb, respectively.

SRPK1 is the main kinase responsible for phosphorylating SR proteins; therefore, the inhibition of SRPK1 activity by TAF15-RGG suggests that the SR protein phosphorylation status might be coordinately altered. We examined this possibility by Western blotting analysis of extracts from HeLa cells transfected with GFP or GFP-TAF15-RGG, using the mAb104 antibody, which recognizes a highly conserved phosphoepitope in SR proteins [34]. We observed that overexpression of GFP-TAF15-RGG resulted in a decrease in mAb104 reactivity, indicating that SR protein phosphorylation was reduced compared to the respective level of phosphorylation in GFP-overexpressing cells (Figure 6A). Since SR protein phosphorylation modulates splicing, we tested the effect of TAF15-RGG on the splicing of a reporter gene. As a reporter gene, we used a rat insulin minigene (pSVIRB) that contains exons 1, 2, and 3 and the respective introns fused with the appropriate SV40 promoter/enhancer regions and insulin transcription terminators, respectively (Fig 6B, upper panel; see also ref. [18]). In this respect, HeLa cells were co-transfected with GFP-TAF-15-RGG and the insulin minigene, and RNA was isolated after 24 h. As shown in Figure 6B, lower panel, in the absence of GFP-TAF15-RGG, the two introns were skipped in about 50%. Overexpression of increasing GFP-TAF15-RGG amounts (0.4, 0.8, and 1.2 μg) resulted in gradual intron retention. No effect was seen in HeLa cells overexpressing GFP alone (data not shown). Co-transfection of FLAG-SRPK1 further increased the percentage of intron skipping, while increasing amounts of GFP-TAF15-RGG resulted in a sharper accumulation of the unspliced pre-mRNA (Figure 6B).

### 3.6. Overexpression of the C-terminal RGG Domain of TAF15 Results in Reduced LBR Phosphorylation

**Figure 7:**
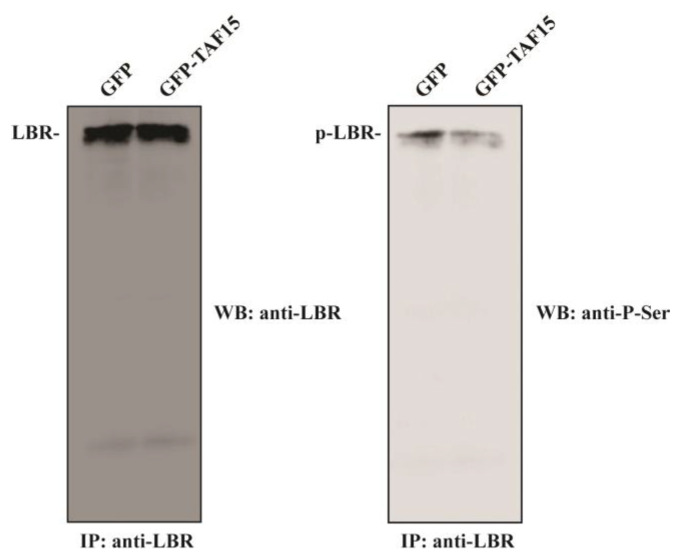
Overexpression of TAF15-RGG results in reduced LBR phosphorylation. HeLa cells were transfected with GFP or GFP-TAF15-RGG. LBR was immunoprecipitated from cell lysates with an anti-LBR monoclonal antibody. Immunoprecipitates were then analyzed by Western blotting using the anti-LBR antibody (**left panel**) and an anti-P-Ser polyclonal antibody (**right panel**).

LBR, a key factor tethering peripheral heterochromatin [35], is another well-characterized substrate of SRPK1 [1]. Therefore, we tested whether the inhibition of SRPK1 activity by TAF15-RGG affected the phosphorylation status of LBR. In this respect, extracts were prepared from HeLa cells transfected with GFP or GFP-TAF15-RGG and immunoprecipitated with an anti-LBR monoclonal antibody. Immunoprecipitates were analyzed on 10% SDS-polyacrylamide gels, and phosphorylated LBR was detected by Western blotting using an anti-P-Ser polyclonal antibody. As shown in Figure 7, overexpression of TAF15-RGG decreased LBR phosphorylation.

### 3.7. Peptides Comprising the RGG Motifs of Nucleolin, HNRNPA2B1, and HNRPU (SAFA) Inhibit SRPK1 Activity In Vitro

**Figure 8:**
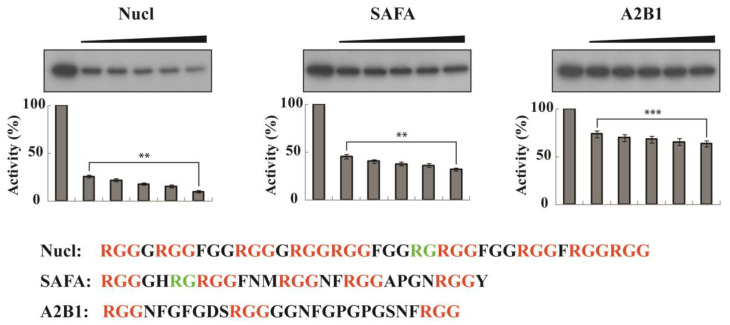
Inhibition of SRPK1 activity by peptides containing RGG repeats. Phosphorylation of GST-LBRNt(62–92) by GST-SRPK1 occurred in the presence of increasing concentrations (0.1, 0.2, 0.3, 0.4, and 0.5 mM) of Nucl, SAFA, and A2B1 peptides, comprising the RGG repeats of nucleolin, HNRPU (SAFA), and HNRNPA2B1, respectively. The sequence of the peptides is shown. RGG are shown in red and RG in green. Data represent the means ± SE of two independent experiments. *p*-values: ** *p* = 0.0072 (for Nucl), ** *p* = 0.00631 (for A2B1), and *** *p* < 3 × 10^−0.5^ (for A2B1).

We have shown that the C-terminal RGG domain of TAF15 binds to and inhibits SRPK1. Similarly, Tunnicliffe et al. [23] have shown that the RGG box motif of ICP27 interacted with SRPK1 and prevented the phosphorylation of the SR protein SRSF1, while the C-terminal domain of SAFB1 (aa 709–915) that could bind to and inhibit SRPK1 activity [21] also contained an RGG/RG-rich domain. Since an RGG/RG domain emerges as the common denominator shared by the three proteins, we then asked whether peptides containing an RGG/RG domain would also inhibit SRPK1 activity. To this end, we used three peptides comprising the RGG motifs of nucleolin, HNRPU (SAFA) and HNRNPA2B1, respectively. These three proteins were previously found to co-immunoprecipitate with SRPK1a, an alternatively spliced isoform of SRPK1 [28]. Nucl (the peptide derived for nucleolin) contained nine RGG repeats and one RG dipeptide, SAFA (the peptide derived for HNRNPU (SAFA)) contained five RGG repeats and one RG dipeptide, and A2B1 (the peptide derived for HNRNPA2B1) contained three RGG repeats and no RG dipeptide (Figure 8A). As shown in Figure 8B, all three peptides could inhibit SRPK1. Most interestingly, the observed inhibition was proportional to the number of RGG repeats, i.e., Nucl exhibited the most potent inhibitory activity and A2B1 the weaker. Hence, RGG-mediated negative regulation of SRPK1 might be a general cellular mechanism for modulating SRPK1 function. 

## 4. Discussion

There are few proteins in the literature that have been characterized as SRPK1 regulators. At first, Sciabica et al. [22] showed that during herpes simplex virus infection, ICP27 was able to associate with and inactivate SRPK1. Next, the nuclear scaffold proteins SAFB1 and SAFB2 were shown to interact with and repress SRPK1 activity [21], and in this study, we present evidence that TAF15 may function as a potent inhibitor of the kinase. All the above proteins, even though they seem functionally unrelated at first sight, harbor RGG/RG motifs. The ICP27 RGG box was shown to directly compete with RS domains for the docking groove of SRPK1, thus preventing phosphorylation of the substrates [23], while the C-terminal domains of SAFB proteins and TAF15 that comprise the RGG/RG repeats were shown to bind to and inhibit SRPK1 ([21], this study). In this respect, it is also worth mentioning that ERH (enhancer of rudimentary homolog protein) was found to interact with the C-terminal region of SAFB1/SAFB2, alleviating the inhibitory effect of SAFB proteins on SRPK1 [36]. The hypothesis that RGG motifs per se may function as potent SRPK1 inhibitors was further strengthened by in vitro phosphorylation assays using three peptides comprising the RGG repeats of nucleolin, HNRPU (SAFA), and HNRNPA2B1, respectively. All three peptides inhibited phosphorylation of the RS domain of LBR, a well-known substrate of SRPK1, and most interestingly, the observed inhibition was analogous to the number of RGG repeats, i.e., the greater the number, the more potent the observed inhibition.

An obvious question that thus arises is why Npl3, which also contains RGG repeats, does not inhibit SRPK1 but, on the contrary, becomes phosphorylated. Yet, Npl3 contains mostly SRGG motifs instead of individual SR/RS and RGG repeats (Figure 1). On this subject, Smith et al. [37] have recently shown that five of the six serine residues (Ser306, Ser328, Ser336, Ser343, and Ser362) in the SRGG motifs of Npl3 were phosphorylated in vitro by Sky1. Npl3 phosphorylation thus seems to depend on the presence of these serine residues and not on the final single RS dipeptide, as initially suggested by Gilbert et al. [27]. The finding that besides phosphorylation of consecutive SR/RS dipeptides, SR protein kinases may also target serine residues within SRGG motifs is further substantiated by the observation that such serine residues in Saccharomyces cerevisiae fibrillarin (Nop1p) and human Cold-Inducible RNA-Binding Protein (CIRBP) were also found to be phosphorylated by Sky1 and SRPK1, respectively [37,38].

Although the steady-state localization of SRPK1 is mostly cytoplasmic and only a small fraction of the kinase is detected in the nucleus, nucleocytoplasmic shuttling of SRPK1 is essential for controlling various cellular processes [39]. Besides post-translational modifications [13,14,18,40], the nucleocytoplasmic shuttling of the kinase seems to also depend on protein-protein interactions inside the nucleus. In this respect, a fraction of SRPK1 is associated with the nuclear matrix in an inactive form due to its binding to SAFB1/2 [21]. In addition, interaction with the viral protein ICP27 resulted in a redistribution of SRPK1 from the cytoplasm to the nucleus [22]. This redistribution was absolutely dependent upon the RGG/RG motif of ICP27 since single point mutations of the arginine residues within the RGG box were sufficient to perturb interaction with SRPK1 and relocalization of the kinase [23]. In line with these previous studies, SRPK1 partially relocalized to the nucleus of cells that expressed TAF15-RGG, showing either diffuse staining throughout the nucleus or nuclear rim fluorescence, whereas the kinase was mainly cytoplasmic in control cells that did not express TAF15-RGG (Figure 5B). SRPK1 phosphorylates in the cytoplasm serine residues on numerous substrates, such as newly synthesized SR proteins, ZO-2, RNF12, and protamine 1, to promote their nuclear import [4,9,10,11,12]. Npl3 nuclear translocation was also dependent on phosphorylation by Sky1 in the cytoplasm of yeast cells [27]. The steady-state localization changes of SRPK1 induced by nuclear proteins containing RGG/RG motifs would therefore lead to a reduction of the kinase levels that are available to interact with its cytoplasmic substrates, thus providing a way to modulate their nuclear entry. On the other hand, the interaction of SRPK1 with RGG/RG-containing proteins would decrease the kinase activity within the nucleus. Accordingly, TAF15-RGG overexpression resulted in hypo-phosphorylation of SR proteins, inhibition of splicing of a reporter minigene, and reduced phosphorylation of endogenous LBR (Figure 6 and Figure 7).

Considerable data exist in the literature to support the physiological relevance of the SRPK1-TAF15 interaction. TAF15 can function as a scaffold protein and drive liquid-liquid phase separation (LLPS) and gelation at low protein concentrations and physiologically relevant salt concentrations [41]. SR protein kinases were shown to impair LLPS via the phosphorylation of SR/SRGG domain-containing proteins. More specifically, SRPK1-mediated phosphorylation of the nucleocapsid protein of SARS-CoV-2 impaired the formation of biomolecular condensates [42], while phosphorylation of the CIRBP SRGG motif suppressed phase separation [38]. In addition, Sky1 was shown to promote stress granule dissolution by phosphorylating Npl3 [43]. TAF15 is known to be methylated on arginine residues in the RGG motif [30]. Methylation of the RGG box of ICP27 at adjacent arginines lowered affinity for SRPK1, and inhibition of the kinase was substantially attenuated [23], while lack of arginine methylation of the RGG repeats of FUS, another member of the FET family, strongly promoted phase separation and gelation [44]. Interestingly, in CIRBP, where both the methylation and phosphorylation sites are located within the SRGG motifs, SRPK1-mediated phosphorylation of serine residues inhibited the methylation of adjacent arginines and vice versa [38]. Similarly, Smith et al. [37] demonstrated that Sky1p-mediated phosphorylation of the SRGG regions in Nop1p blocked arginine methylation by Hmt1p.

The microprocessor complex that mediates the genesis of microRNAs is a paradigm of a multiprotein complex comprising an RS domain-containing protein, an SRPK, and TAF15 [45]. Drosha, an RNAse III endonuclease mainly responsible for miRNA processing, contains a significant number of SR/RS dipeptides within aa 245–360 and, therefore, could be targeted by SRPK1a, an alternatively spliced isoform of SRPK1, which was also identified as a component of the microprocessor complex. TAF15, as well as FUS and EWS, the other two members of the FET protein family, are also members of the complex. It is currently unclear whether components of the microprocessor complex can phase separate, indicating a possible connection between LLPS and miRNA biogenesis, nor the influence of the phosphorylation state of the SR/RS dipeptides on Drosha interactions with RNAs and the other members of the complex. Yet, there are reports on the involvement of TAF15 [46], FUS [47], and EWS [48,49] in the regulation of pri-miRNA processing. Interestingly, SAFB2 has also been recently described as an accessory protein of the microprocessor with a role in pri-miRNA processing [50].

Thus, SR/RS domain phosphorylation mediated by SRPKs and negative regulation of the kinases by RGG repeats may have developed as an effective competitive strategy to regulate various cellular processes. Further research is required to elucidate whether this is ubiquitously achieved via fine-tuning the reversible compartmentalization of specific proteins and RNAs into lipid droplets and shed light onto the biophysical and biochemical principles that determine the interactions between SR protein kinases, RNA, and RS and RGG domain-containing proteins and the role of post-translational modifications on these interactions.

## Data Availability

Not applicable.

## Supplementary Materials

The following supporting information can be downloaded at: https://www.mdpi.com/article/10.3390/cells12010126/s1, Figure S1: The N-terminal part (aa 1-320) of TAF15 has no effect on SRPK1 activity.
