# Supplementary material for: SR Protein Kinase 1 Inhibition by TAF15
Source: Cells. 2022 Dec 28;12(1):126. doi: 10.3390/cells12010126 (PMC9818988; doi:10.3390/cells12010126)
Supplement: Supplementary file 1 [file cells-12-00126-s001.zip › cells-2097278-supplementary.pdf]

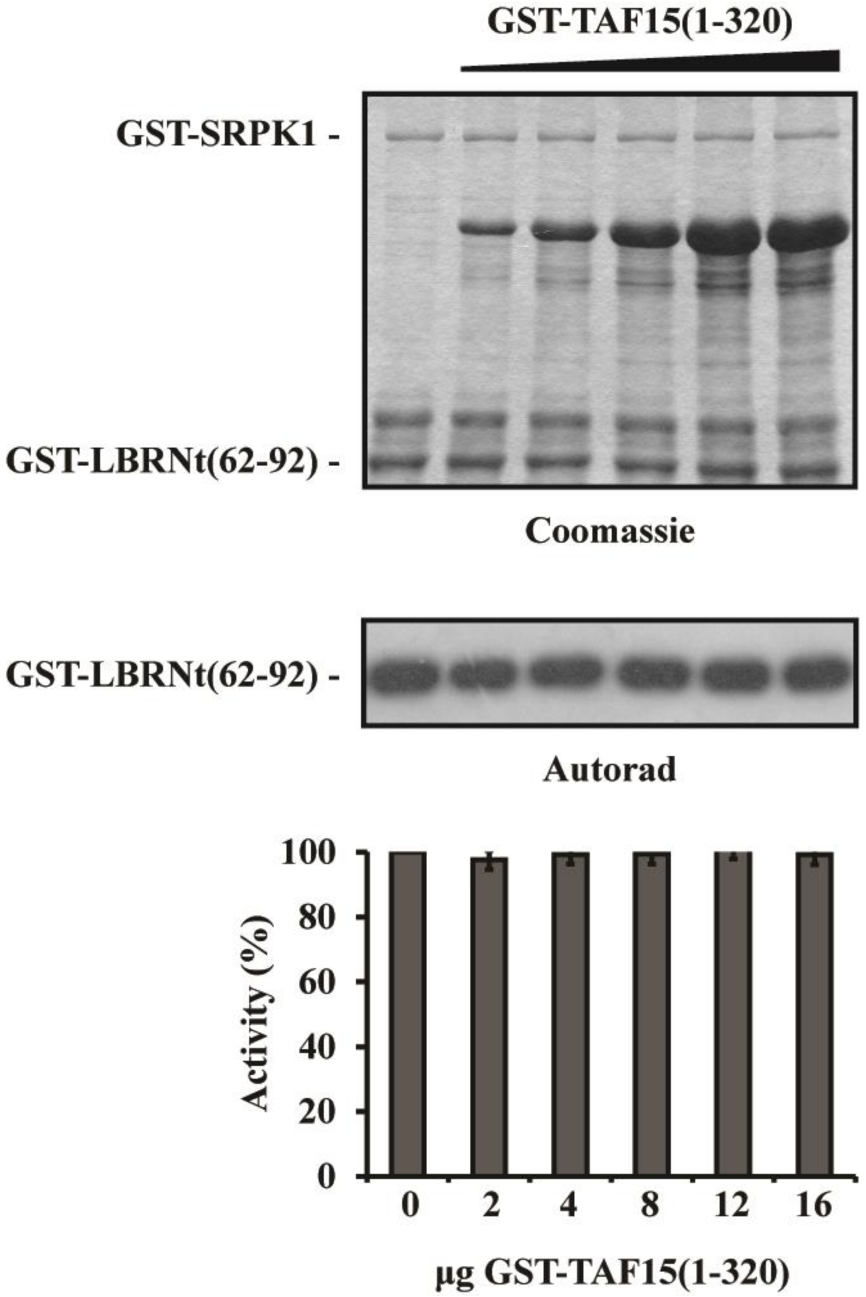

Figure S1. The N-terminal part (aa 1-320) of TAF15 has no effect on SRPK1 activity. GST-SRPK1 kinase was incubated with GST–LBRNt(62-92) and [γ-32P]ATP in the presence of 2, 4, 8, 12 and 16 µg GST-TAF15(1-320). The samples were analyzed by SDS-PAGE, Coomassie blue stained (upper panel) and autoradiographed (middle panel, only the respective part of the gel is shown). Phosphorylated bands were excised from the dry gel and Cherenkof counted (lower panel). Data represent the means ± SE of two independent experiments.
